# Editorial: The built environment and public health: New insights

**DOI:** 10.3389/fpubh.2022.1079182

**Published:** 2023-01-04

**Authors:** Linchuan Yang, Ruoyu Wang, Baojie He, Yu Ye, Yibin Ao

**Affiliations:** ^1^Department of Urban and Rural Planning, School of Architecture, Southwest Jiaotong University, Chengdu, China; ^2^UKCRC Centre of Excellence for Public Health/Centre for Public Health, Queen's University Belfast, Belfast, United Kingdom; ^3^Centre for Climate-Resilient and Low-Carbon Cities, School of Architecture and Urban Planning, Chongqing University, Chongqing, China; ^4^Department of Architecture, College of Architecture and Urban Planning, Tongji University, Shanghai, China; ^5^College of Environment and Civil Engineering, Chengdu University of Technology, Chengdu, China

**Keywords:** physical environment, living environment, physical health, psychological health, healthy city, wellbeing, quality of life, life satisfaction

## Background

According to “World Cities Report 2022: Envisaging the Future of Cities,” 56% of the global population is currently living in cities, which is expected to reach 68% by 2050. The rapid growth of cities led to the emergence of various problems, such as environmental pollution, traffic congestion and emissions, and the lack of physical activity of residents (1). Such problems prompted a widespread public focus on the urban environment and health issues. According to World Health Organization estimates, about 24% of the global public health burden can be attributed to the changing environment. Thus, integrating public health issues into the sustainable development of a city has become a significant issue around the world.

The built environment, which is a subset of the physical environment, interacts with public health in an intricate way. This relationship, which is highly pertinent to policymaking, fostered interesting academic explorations in recent years (2–4). However, based simply on traditional data and approaches, many relevant issues remain unexamined (5). With the rapid development of science and technology, accurate built environment/health metrics can be assessed using open/big data or advanced instruments, advanced modeling approaches can be tested and applied to the research field, new/traditional theories can be supported or refuted with strong empirical evidence, and rich insights into policy and practice can be discussed with highly consistent or contradictory findings, especially during and after the coronavirus disease (COVID-19) pandemic (6–8). Hence, propelling research on the association between the built environment and public health is urgent and vital. Such research can provide us with a comprehensive understanding and profound implications.

In light of the above discussion, we launched this Research Topic in *Frontiers in Public Health* and announced a “call for papers” in August 2020. After a rigorous peer review, a total of 43 articles–including three literature reviews and 40 research articles–were published on this Research Topic, with 193 researchers as the authors of the papers. The collection of articles on this Research Topic was completed in September 2022.

This Research Topic is a platform for sharing fresh insights and findings on the association between the built environment and public health and an essential and enriching memo to decision-makers, health researchers, caregivers, and so on. We aim to raise awareness of all the elements of society (e.g., governments, researchers, businesses, industry, and individuals) and stimulate wide, in-depth discussions on the association between the built environment and public health in developed and developing countries. Moreover, we call for collective action by all the members of society as the world strives to achieve the United Nations' Sustainable Development Goals, which entails consensus, collaboration, and innovation.

## Research Topic contributions

### Theme 1: COVID-19

The COVID-19 pandemic has affected the world profoundly for nearly 3 years. The pandemic attracted the attention of numerous researchers from diverse disciplines worldwide (9). Six papers in this collection focus on COVID-19-related issues.

Frontline nurses play a vital role in combating COVID-19. However, nurses suffered from work-related fatigue owing to increased workloads during the COVID-19 pandemic, which caused their health to deteriorate and affected their safety. Ma et al. explored the connection between the environmental factors affecting infectious disease nursing units and nurses' fatigue. The analysis results showed that nursing distance is the most important variable affecting nurses' physical fatigue, whereas spatial privacy is the dominant factor influencing their psychological fatigue. Environmental factors are related more to physical fatigue than psychological fatigue, and the job position of nurses is significantly associated with the perception of fatigue. Therefore, the authors argued that such factors (e.g., shortening the nursing distance and enhancing spatial privacy) should be considered to alleviate fatigue.

Mass vaccination is an effective means of combating COVID-19. Wu, Ming, et al. investigated vaccination intention and associated factors among individuals with HIV. This study confirmed that individuals with HIV demonstrate more severe vaccine hesitancy than the general public, and many factors, such as physical condition, perceptions, and economic factors, influence willingness to receive COVID-19 vaccination.

The risk of the spread of COVID-19 should be minimized to reduce the number of casualties and property losses. Duan et al. explored the risk elements and risk transmission paths of COVID-19 as an example of a sudden major infectious epidemic. The author obtained 20 initial concepts, 11 categories, and six main categories and found that (1) 6 risk transmission elements for major infectious epidemics as well as (2) 3 risk transmission pathways for sudden infectious diseases, namely the transmission pathway of the medical system, transmission pathway of the social system, and transmission pathway of the psychological system. Based on the results, the author suggested various measures for the emergency management of sudden epidemic outbreaks, such as establishing a scientific event risk early warning system.

Poor urban management is widely observed when addressing major public health emergencies such as COVID-19. Wu, Chen, et al. took Wuhan as a case and analyzed the problems in urban management during the COVID-19 pandemic. The authors selected five basic urban management modules and identified the shortcomings of each one in combating the pandemic. Finally, the authors proposed a battery of key strategies for post-pandemic urban revitalization.

Housing is part and parcel of people's lives. Thus, it can be reasonably expected that the COVID-19 pandemic will lead to changes in people's housing needs. Xu and Juan presented a systematic study on design strategies for apartments in China in the post-pandemic era and compiled a set of design strategies. The authors identified the respondents' preference for the quality attributes of apartments through a questionnaire survey in three cities in Jiangsu Province, China. The authors found that (1) men need more social space than before, (2) older adults have little need for office space and high expectations of the inclusion of an ensuite bathroom in the primary bedroom, and (3) a significant positive association exists between education level and need for office space. Notably, this work contributes to important custom decisions for design firms, housing developers, and the government in planning and strategizing China's apartment construction in the post-pandemic era. In a departure from the above study focusing on apartment design strategies, Guo X. et al. analyzed the public's willingness to pay for healthy housing. The authors proposed a theory of planned behavior-based model and conducted partial least squares structural equation modeling using eye-tracking and questionnaire data. This study unraveled the complex relationship between information attentiveness, attitude, subjective norm, perceived behavioral control, and people's willingness to pay for healthy housing.

### Theme 2: Multi-dimensional health

With the improvement in living standards, people have become increasingly concerned about their health. Eight papers in this collection pay attention to multi-dimensional health.

The neighborhood environment was extensively confirmed to be an essential determinant of residents' health and life satisfaction. Chen et al. focused on the impact of the neighborhood environment on three health outcomes (i.e., frailty, activities of daily living, and instrumental activities of daily living) of 969 frail older adults and conducted correlation analysis and path analysis on the examined problem. Moreover, the authors treated quality of life and service use as intermediate variables. The findings demonstrated that the neighborhood-level built environment has a significant impact on an individual's quality of life and health. The deterioration of older adults' health conditions can be mitigated through the provision of accessible services and groceries, high metro accessibility, and exposure to greenery. The findings can inform policymakers and stakeholders to increase access to services and transportation and greenery planning. Similarly, Li J. et al. established an analytical framework for the association between the neighborhood-level built environment and the health of older adults (or senior health). The authors developed multilevel regression and structural equation models using large-scale official data (“Fourth Survey on the Living Conditions of the Elderly in China”) and street-view imagery data for empirical analysis. Notably, the authors controlled for residential self-selection. Finally, some strategies for improving aging health were proposed from the perspective of urban planning. Meanwhile, Liu J. et al. used structural equation models to confirm the significant influence of the perceived neighborhood environment on health behaviors, health outcomes, and life satisfaction. The authors observed a substantial moderating effect of sociodemographics on the above relationships. Policymakers can benefit from such nuanced empirical evidence in implementing targeted and tailored interventions.

A pleasant natural or built environment can help maintain individuals' satisfactory state of mental health and reduce the possible emergence of psychological problems. The creation of such an environment has become the main focus of the government and academia. Han X. et al. proposed a novel method for predicting the perceived psychological stress of residents based on street-view imagery and machine learning and employed the method in Seoul, South Korea. The authors found that the perception of psychological stress is spatially autocorrelated in the city. Specifically, various land-use functions have a significant influence on perceived psychological stress. The results of this study can enhance our understanding of human-environment interactions. Meanwhile, Yang, Cui, et al. focused on the mental health of undergraduates and conducted a battery of linear regressions to explore the association between a campus-centered natural/built environment and depression among undergraduates in 89 campuses in various regions of China. Moreover, the authors examined the moderating role of socioeconomic attributes. The results showed that scattered campuses are negatively associated with undergraduates' depression, whereas campuses with numerous outlets serving takeaway sweets and fast food were positively related to the undergraduates' depression. Thus, optimizing the campus environment can potentially relieve undergraduates' depressive moods and promote their mental health. Furthermore, as a special demographic group, children are highly sensitive to the environment. Fu F. et al. investigated the mental health of troubled children in an SOS children's village community in Chengdu using the PHCSS-SD method and identified four types of space for important outdoor activities. This study can contribute to the development of child-friendly communities.

In stark contrast to the above micro-scale studies, two studies investigated health issues from a macro-scale perspective. First, environmental pollution and economic growth are significant factors affecting public health. Zhao et al. analyzed the relationship between environmental pollution, economic growth, and public health in 30 provinces and cities in China from 2007 to 2018. The authors also scrutinized the differences in the impact of environmental pollution and economic growth on public health in the eastern, northeastern, central, and western regions of China. Second, the Chinese-American population is growing rapidly. Zhang Q. et al. conducted a spatial analysis of Chinese-American ethnic enclaves in New York (the United States). In addition, the authors modeled the correlation between residents' propensity to live in a Chinese-American enclave and two health indicators (i.e., access to health care and perceived health status) using a logistic regression model and observed significant correlations.

### Theme 3: Physical activity

Physical activity, as a type of health behavior, is an interesting and meaningful topic. Top academic journals such as *Nature* and *The Lancet* have published papers related to this topic (10–16). Eight papers in this collection focus on physical activity.

The outdoor physical activity of the general public was studied by several scholars. Li B. et al. investigated the impact of the built environment on the frequency and intensity of residents' outdoor physical activities (i.e., walking and running) in Changsha (China). Based on data extracted from wearable devices and various open data sources, the authors developed a set of linear regression models to scrutinize the connection between the built environment and a set of outdoor physical activity variables. Similarly, Yang, Yu, et al. used data from Strava to derive two outdoor physical activity indices (i.e., running and cycling) and analyzed the association between these indices and the built environment of Chengdu, which is a megacity in southwest China, using spatial econometric methods. The authors obtained rich findings and proved the applicability of crowdsourced data in physical-activity research. Notably, the authors analyzed many built-environment attributes rarely examined in the literature, such as river line length and the light index.

Population aging has become a demographic trend in recent decades, and physical activity is arguably an essential aspect of healthy aging. Therefore, the physical activity of older adults has received widespread scholarly attention. Zang et al. examined the heterogeneity in the association between walking time and the built environment in Guangzhou (China) and modeled the environmental characteristics of three zones with different building densities (low-, medium-, and high-density zones). The authors mainly considered (1) differences between recreational walking and transport walking and (2) differences in areas with varying building densities. Built-environment attributes in buffer zones within different radius ranges were identified in the GIS. The results of this study confirmed the existence of heterogeneity in the relationship. Moreover, the neighborhood is believed to be able to influence elderly adults' health and quality of life in many ways (e.g., by affecting physical activity levels). Liu Z. et al. investigated the association between the neighborhood and physical activity, health, and quality of life of older adults in a comprehensive framework. The study results showed that social capital contributes to health and quality of life through participation in leisure-time physical activity. This knowledge has important implications for urban planners and policymakers to create livable neighborhoods to promote healthy aging. Similarly, Xiao et al. explored how physical activity mediates the relationship between the built environment and obesity (measured by the body mass index) among older people. As evidence from the literature is weak, the authors hypothesized that an environment that is friendly to older adults could encourage them to walk more. As a result, the authors conducted a bootstrap mediation analysis to examine the mediating role of exercise. Finally, the authors found that calorie expenditure-related facilities, such as gyms, parks, and grocery stores, can significantly encourage older adults' physical activity, thereby reducing their risk of obesity.

Riding a shared bike has become a popular physical activity, especially in recent years. Considering the proliferation of bike share services, Guo Y. et al. presented a systematic review of the relationship between bike share usage (one type of physical activity) and the built environment. Instead of solely discussing/identifying the built-environment attributes associated with bike share usage (e.g., land-use mix, urban amenities, and transit accessibility), the authors paid considerable attention to variances in the relationship between bike share usage and the built environment, which can be explained by different study areas, bike share services (docked vs. dockless), time of day (morning vs. afternoon vs. evening), day to week (weekdays vs. weekends), and so forth. The authors also identified several avenues for further research, such as diversifying the study area, shifting to the perceived built environment, and delving into the interaction between bike share usage and the metro. Notably, this work contributes to explaining the controversy in existing findings on the connection between bike share usage and the built environment.

A pedestrian-friendly environment can help promote people's walking behavior. Jin et al. a revealed how the micro-level environment affects people's walking behavior. The author used an unobtrusive tracking method to collect the walking routes of hundreds of residents and developed a discrete choice model (specifically, the conditional logit model) to scrutinize the environmental factors influencing pedestrians' perceptions and walking route preferences. The authors discovered that various features, such as sidewalks, driveways, and trash cans, affect people's walking behavior. Similarly, Jin et al. b used binary logistic regression models to investigate the interaction between the street environment and people's route choice behavior and found that many street elements, such as parking, trash cans, and street lights, significantly influence pedestrians to choose the non-shortest routes. Furthermore, the authors revealed potential correlations between network attributes, personal factors, and trip characteristics.

### Theme 4: Physical environment

The physical environment permeates every aspect of our lives. It not only affects people's physical health but also has an important implication on people's psychological health. Many scholars conducted research on different aspects of the physical environment. Five papers in this collection focus on the physical environment.

The physical environment is a multi-dimensional concept (including but not limited to sound, wind, heat, and light) and is closely linked with public health. Wu Y. et al. assessed the health-related physical environment of a university campus in Hangzhou (China) in terms of the following four dimensions: sound, wind, heat, and landscape. Based on the evaluation outcomes, the authors suggested five methods for spatial optimization, including creating ventilation corridors and applying cooling measures. Notably, this study identified priority areas for intervention in spatial planning and design.

Air is an important part of the physical environment. Air pollution can seriously endanger the health of urban residents and lead to a wide range of diseases; thus, demand for satisfactory air quality has become increasingly intense. Two studies analyzed the implications of air pollution/quality in Chinese cities for socioeconomic systems. Lai et al. used a geographically weighted regression model to estimate the marginal willingness of housing buyers to pay for clean air in different urban areas. The results showed that people are more willing to pay for clean air in areas with more serious pollution (e.g., streets with heavy traffic congestion and places near heavy industrial factories). In addition, the authors suggested that the government can improve the urban energy structure and decrease industrial exhaust emissions according to different taxes and policy interventions among regions to improve urban air quality. The study can help the government analyze the cost-benefit tradeoff of air pollution control. Meanwhile, Li D. et al. evaluated the health benefits of air quality improvement in a provincial capital city in western China to test the causal relationship between particulate matter that is 2.5 microns or less in diameter (PM_2.5_) and residents' medical expenditures. The authors determined that with improved air quality resulting from substantial environmental investments, local residents can obtain high health benefits. In other words, residents situated in harmful surrounding areas may encounter health risks to a certain extent. The regression results demonstrated that the medical expenditures of patients with high reimbursement rates are impacted heavily by air pollution. Therefore, the government should optimize the social budget at a synthetic scope. In addition, environmental contaminants with additional costs should be highlighted by different government sectors.

In a departure from the above two studies focusing on the implications of air pollution/quality for socioeconomic systems, one study evaluated the effects of air pollution/quality on the physical environment. Zhang X. et al. aimed to analyze the effect of air pollutants on solar radiation and used nine Chinese cities as the study area. Notably, this study filled a research gap and pioneered research on the correlation between individual pollutants and daily global solar radiation and its spatial variability.

Reasonable lighting design can ensure driver safety in long tunnels. The research of Peng et al. was dedicated to exploring the relationship between drivers' psychological and physiological states and changes in the lighting environment. The authors investigated the characteristics of drivers' physical fatigue and mental load and changes under perceived low ambient luminance, changing the roadway luminance or perceived ambient luminance conditions in long tunnels through field tests. The study also investigated the reasons behind the relief of physical fatigue and mental load in the case of increasing contour sections. Finally, the authors focused on methods for improving the lighting environment and provided new ideas on the lighting design of the inner zone of long tunnels.

### Theme 5: Indoor environment

People spend most of their time indoors. A healthy and comfortable indoor environment can induce satisfactory life experiences. Therefore, the quality of the indoor environment has attracted increasing attention. Three papers on this Research Topic focus on the indoor environment.

The enhancement of the indoor environment of public buildings may be conducive to people's physical and psychological needs and health. Fang et al. revealed evacuation optimization strategies for two types of large-scale public building, namely stadiums and high-rise hospitals. To minimize personnel's time in the process of safe evacuation and enhance evacuation efficiency for the whole building, they proposed two strategies for plane evacuation and vertical evacuation. In addition, the authors developed a stadium model and a high-rise hospital model to verify the effectiveness of the proposed strategies. Unlike the above study on large-scale public buildings, Wang Z. et al. focused on the health and thermal comfort issues of unit-type student apartments in western China through field measurements and a questionnaire survey. Besides traditional field measurements and survey data analysis, the authors analyzed the correlation between thermal comfort-related variables in the summer and winter separately. The study advanced understanding of the factors significantly correlated with thermal sensations.

In addition to the above two empirical research papers, a literature review paper was included on this Research Topic. Zhou Y. et al. concentrated on the value-based design of healthcare facilities (indoor environment). The author extracted and analyzed nearly 500 papers published in representative American and Japanese journals. Based on the literature review, the authors proposed a novel conceptual framework for the value-based design of healthcare facilities. Notably, the authors emphasized the importance of the introduction of three value variables (i.e., time, space, and behavior), which is beneficial to research and practice.

### Theme 6: Transport

Many scholars examined transport-related issues (e.g., pedestrian behavior, transportation demand management, and traffic pollution and emissions) to make transport effectively serve society. Five papers in this collection focus on transport.

Accurate traffic flow forecasting is fundamental to efficient and effective transport management. Yao et al. proposed a deep learning-based traffic flow prediction framework consisting of various components, each of which includes a spatial block and a temporal block. The framework performs fewer calculations to obtain more accurate prediction outcomes than state-of-the-art baselines. The study also identified several avenues for further research, such as integrating other factors into the model to improve its prediction accuracy.

Taxis are a popular public transport mode in many cities. The modeling of the customer-search behavior of taxi drivers is an intriguing research topic. Yu L. et al. mined multiple GPS records of 8,400 taxis in Shanghai (China) to identify the determinants of taxi drivers' customer-search behavior *via* time-dependent discrete choice modeling. The authors examined the role of relative passenger demand, regional pickup likelihood, expected rate of return, en-route delay, and traffic conditions at target hotspots in shaping customer-search behavior in various periods. The study can provide a basis for service providers and policymakers to optimize taxi services, thereby contributing to balancing the spatiotemporal mismatch between taxi demand and supply.

Car-sharing flourished in the last two decades. Dashboard design is a factor considerably affecting the experience of car-sharing users (drivers). Yang, Wang, et al. explored the relationship between users' driving activities and dashboard layout using the experimental investigation and prediction approach, established a user heart rate prediction model, and conducted a set of correlation analyses. The authors pointed out that the system usability of a dashboard is correlated with various eye-tracking characteristics. Notably, this work can help optimize the selection of appropriate dashboard layouts in interface design in the future.

Transit-oriented development (TOD) is a desirable and favored development pattern, especially in megacities. However, seldom has its performance been evaluated comprehensively. Qiang et al. innovatively evaluated TOD performance in three ways [i.e., transportation (T), pedestrian-oriented accessibility (O), and urban development (D)] using multi-source data. The authors chose Shanghai as the study area and assessed 347 metro station areas using clustering and correlation analyses. The results help advance our understanding of TOD performance and benefit policymakers, urban planners, researchers, and so on.

Energy consumption and emissions in the transport sector were extensively evaluated using simulations, statistical analysis, machine learning, mathematical programming, and so on. Wang W. et al. used real-world GPS trajectory data to evaluate the influence of a regional speed control strategy on energy consumption and traffic emissions in Beijing (China). The authors provided suggestions on residents' living activities, such as taking precautions while jogging and avoiding jogging on urban arterial roads. The study also identified several avenues for further research, such as collecting increased data and details of vehicle driving data to improve the accuracy of emissions calculation.

### Theme 7: Hospitals

A hospital is a type of healthcare institution, essential for healthy city development. Three papers in this collection focus on hospitals.

Yu J. et al. investigated access to primary hospitals among older adults using the two-step floating catchment area method and evaluated their satisfaction with the access using a questionnaire survey. The authors further examined the association between the two factors, and the results indicated that the distribution of primary hospitals is unequal. Older adults' satisfaction with hospitals is significantly influenced by the walking distance to the hospitals. Specifically, satisfaction with hospitals decreases with increasing walking distance.

Liu Y. et al. focused on the reputation crisis management level of hospitals. They developed a reputation index and crisis respondent index, and a quadrant approach for classification. The authors empirically assessed five hospitals using the proposed method and categorized the reputation positioning of the hospitals into four groups, namely robust, growth, fragile, and sensitive. Based on the results, the authors proposed strategies for hospital reputation crisis management.

Cui et al. assessed hospital rest spaces and presented a micro-scale study. The authors investigated differences in the eye movement measures of staff with different attributes and the correlation between the eye movement measures and scores on the self-rating restoration scale. Accordingly, the authors proposed a set of key points in the planning and design of hospital outdoor rest spaces, such as setting up outdoor rest spaces near work areas, paying attention to the safety needs of senior staff and work pressure of junior nurses, and improving brightness, saturation, and access to the sky environment.

### Theme 8: Public space quality

Public spaces (e.g., public squares, parks, streets, and beaches) have been regarded as an important part of contemporary urban design because their quality has a significant impact on people's behavior and health. Therefore, examining the relationship between public space quality and people is imperative to provide the latter with high-quality public spaces. Two papers in this collection help to bring further exploration in this direction.

Public open spaces have profound implications for outdoor environment exposome and public health. Han S. et al. presented a systematic review of interactions between people's behavior and the built environment in public open spaces and highlighted the impacts of diverse and objective influencing factors. By selecting 109 representative studies on people's behavior in public open spaces based on the PRISMA flow diagram, behavioral influencing processes, including objective factors, subjective feedback, and the relationships involved, were considered to provide a comprehensive picture. The authors stressed the importance of four types of factor (i.e., personal background, location and context, environmental components, and climate stimuli) and the health impacts of people's outdoor behavior. With the robust classification of the existing factors, architects, urban designers, policymakers, and researchers can get a comprehensive trend from the past.

One study focused on a specific type of public place. Based on the principles of humanistic urban planning, Wan et al. measured street space quality from the perspective of pedestrians. The study consisted of two parts: (1) construction of a relatively comprehensive “5D + 3S” spatial quality measurement model for streets and (2) testing of the validity of the model. The results showed a high degree of consistency between the subjective and objective measurements, thereby verifying the validity and rationality of the model. The study provides a new way of thinking about the large-scale comprehensive diagnosis of street space quality and can help guide space design through the street space quality measurement results.

### Theme 9: Rural areas

The importance of rural areas is considerably emphasized in the advocacy of urban-rural integration and rural revitalization. Recently, an increasing number of scholars have devoted themselves to rural area research. Three papers in this collection focus on rural studies.

As a public space that is important to people's daily lives, streets are gradually drawing the attention of health environment researchers. Fu E. et al. conducted laboratory experiments on two forms of high human senses (i.e., visual and auditory) to investigate the healing potential of street environments in rural communities. The authors revealed that street type could substantially affect its healing potential, and artificial–natural enclosed and natural semi-enclosed streets have the best healing effect.

Rural infrastructure is the basis for ensuring the livelihood of rural residents, and sanitation is one of the basic services closely related to health. Against this background, Wu, Zhang, et al. examined residents' willingness to pay for and participate in sanitation improvement in rural areas in western China. The authors explored the factors influencing such decisions from the perspective of demographic characteristics, personal interests, living environments, and sanitation conditions. The study supported the design of related public-private partnership projects. Zhou W. et al. focused on another rural infrastructure (i.e., the rural water supply system, RWSS) and its seismic resilience. The authors obtained over 40 potential factors affecting the seismic resilience of the RWSS through a systematic literature review and semistructured expert interviews and conducted an empirical analysis in Sichuan Province, China. The findings showed that “economic resilience” and “organizational resilience in the disaster prevention stage” are the most important influencing factors. Finally, different experiences in earthquake disasters facilitate stakeholders' different perceptions of the importance of such factors.

## Conclusion

As people pay increasing attention to public health, especially during the COVID-19 pandemic, promoting research on the interaction between the built environment and public health is important and indispensable. Many future research directions can be envisioned, including but not limited to the use of new fine-grained data (e.g., street greenery, wearable sensors, and eye-tracking system) to assess environmental exposure and/or health outcomes, advances in the frontier theory of the built environment and health (BE–health) relationship (e.g., spatial life-course epidemiology, effects of new factors such as bike share on public health, the use of new modeling approaches (e.g., machine learning techniques) to elucidate BE–health relationship, BE–health studies in underexamined areas (e.g., cities and countries in the Global South), the nonlinear and threshold effects of the built environment on health outcomes, and socioeconomic inequalities in the BE–health relationship and its implications (e.g., equigenesis theory).

This Research Topic assembles 43 research papers examining the association between the built environment and public health from multiple perspectives and provides new insights that can help enrich and strengthen our understanding. We hope that other studies that can advance our understanding will be conducted in the near future.

## Author contributions

All authors listed have made a substantial, direct, and intellectual contribution to the work and approved it for publication.
